# The complete mitochondrial genome of *Dryopteris crassirhizoma* Nakai (Dryopteridaceae, *Dryopteris* Adanson)

**DOI:** 10.1080/23802359.2021.1966344

**Published:** 2021-08-18

**Authors:** Yue-Yue Song, Xu-Sheng Cui, Liang Xu, Yan-Ping Xing, Che Bian, Yang Qiao, Yan-Yun Yang, Ting-Guo Kang

**Affiliations:** a School of Pharmacy, Liaoning University of Traditional Chinese Medicine, Dalian, PR China; b Shijiazhuang Yiling Pharmaceutical Co., Ltd, Dalian, PR China

**Keywords:** Complete mitochondrial genome, phylogenetic tree, *Dryopteris crassirhizoma*

## Abstract

The complete mitochondrial genome of *Dryopteris crassirhizoma* was sequenced for the first time. The mitochondrial genome length was 313,346 bp, with 48.58% GC contents. There were 94 genes annotated, including 27 known protein-coding genes, 49 *tRNAs*, and 18 *rRNAs*. The maximum likelihood method was used to establish the phylogenetic tree of six species. The phylogenetic results showed that *D. crassirhizoma* was sister to *Ophioglossum californicum.* It reveals the genetic relationship between different species and provides a theoretical basis for the establishment of a classification system.

*Dryopteris crassirhizoma* is a plant belonging to Dryopteridaceae, whose rhizome and petiole residues are used as Chinese medicinal materials, which are known as ‘Mianmaguanzhong’ . Three provinces of Northeast China and their surrounding areas are its main distribution areas, and Jilin province of China is its genuine producing area (Zhu et al. 2017). The main chemical components of Mianmaguanzhong are phloroglucinols, terpenes, flavonoids, sterols, and volatile oils, etc., and the pharmacological effects of that are multiple, including antiviral, antibacterial, anthelmintic, and hemostatic (Wang et al. 2020). Nowadays, a large number of its adulterants are circulating in the market, resulting in the complex composition of its medicinal plants. Many scholars have more accurately identified the original plants of medicinal materials by analyzing its sequence using molecular identification, *psbA-trnH* sequence (Cai et al. 2016) and plastid genome (Xu et al. 2019), etc.

Fresh leaves of *D. crassirhizoma* were collected from Benxi City, Liaoning Province, China (E 124°36′, N 41°15′). The voucher specimen was identified by Professor Ting-guo Kang (Liaoning University of Chinese Medicine, Dalian, China). The voucher specimen and genomic DNA were deposited at the herbarium of Liaoning University of Chinese Medicine (Liang Xu 861364054@qq.com, *D. crassirhizoma* number: 10162201128089LY). Total genomic DNA was extracted from fresh leaves with a Plant Tissue Mitochondrial DNA Extraction Kit (Genmed Scientific Inc., Arlington, MA) and sequenced by Illumina NovaSeq 6000 and Nanopore platform (Zhang et al. 2021). Assemble the sequencing data with GetOrganelle version 1.7.1 (http://github.com/Kinggerm/GetOrganelle. USA) (Jin et al. 2020) and correct the bases with Pilon version 1.23 (http://github.com/broadinstitute/pilon. USA) to get the final genome sequence of mitochondrion. The protein-coding sequences of mitochondria were compared with known protein databases (NR, Swiss-Prot, eggNOG, KEGG, GO) to predict protein-coding genes.

The mitochondrial genome of *D. crassirhizoma* was 313,346 bp in total length with the typical circular structure, and GC content was 48.58%. There were 94 genes annotated, including 27 known protein-coding genes, 49 *tRNAs*, and 18 *rRNAs*. The accumulated length of the coding gene was 20,928 bp, and the length of the coding region accounted for 6.68% of the genome. In addition, we found that seven genes (*cox1*, *nad7*, *nad2*, *cox2*, *nad4*, *nad1*, and *nad5*) contained 28 introns.

RNA editing appeared in the sequencing of mitochondrial genome. RNA editing exists widely in the plastids and mitochondria of Bryophyceae, Lycopodiaceae, and ferns, but its range and mechanism still need to be further study. There are only two known mitochondrial genomes from fern in the GenBank, *Ophioglossum californicum* and *Psilotum nudum* (Du et al. 2019; Wan 2013) . We selected the complete mitochondrial genome of six species (including *D. crassirhizoma*) to construct the phylogenetic tree, using the maximum-likelihood method with the model GTR + I + G (Figure 1). Among them, *Ahnfeltia plicata* and *Andreaea wangiana* as the outgroup. The phylogenetic tree showed that six species clustered to two clades, and *D. crassirhizoma* was sister to *O. californicum* and *A. plicata* and *A. wangiana* are far from the other species. The results are consistent with the cladistic relationship described in the PPG I fern system and the Chinese vascular plant tree of life (Adjie and Lestari 2014). These data will provide a basis to analyze the phylogenetic position of *D. crassirhizoma*.

**Figure 1.**
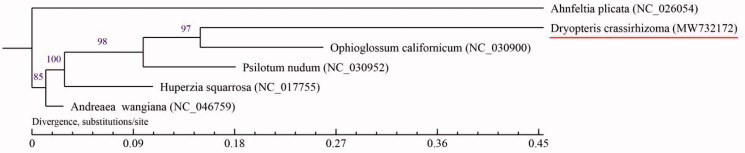
Maximum-likelihood (ML) phylogenetic tree of *D. crassirhizoma* and five other species. Numbers above the branches indicate the bootstrap values from ML analyses.

## Data Availability

The genome sequence data that support the findings of this study are openly available in GenBank of NCBI at (https://www.ncbi.nlm.nih.gov/) under the accession NO. MW732172. The associated BioProject, SRA, and Bio-Sample numbers are PRJNA698451, SRX10003060 (OXFORD_NANOPORE), SRX10003059 (ILLUMINA), and SAMN17719000, respectively.
